# Spontaneous Production of Immunoglobulin M in Human Epithelial Cancer Cells

**DOI:** 10.1371/journal.pone.0051423

**Published:** 2012-12-12

**Authors:** Fanlei Hu, Li Zhang, Jie Zheng, Ling Zhao, Jing Huang, Wenwei Shao, Qinyuan Liao, Teng Ma, Li Geng, C. Cameron Yin, Xiaoyan Qiu

**Affiliations:** 1 Department of Rheumatology and Immunology, Peking University People’s Hospital, Beijing, China; 2 Department of Immunology, School of Basic Medical Science, Peking University, Beijing, China; 3 Department of Gynaecology, Peking University Third Hospital, Beijing, China; 4 Department of Hematopathology, The University of Texas MD Anderson Cancer Center, Houston, Texas, United States of America; Karolinska Institute, Sweden

## Abstract

It is well known that B-1 B cells are the main cell type that is responsible for the production of natural immunoglobulin M (IgM) and can respond to infection by increasing IgM secretion. However, we unexpectedly found that some epithelial cells also can express rearranged IgM transcript that has natural IgM characteristics, such as germline-encoded and restricted rearrangement patterns. Here we studied IgM expression in human non-B cells and found that IgM was frequently expressed by many human epithelial cancer cells as well as non-cancer epithelial cells. Moreover, CD79A and CD79B, two molecules that are physically linked to membranous IgM on the surface of B cells to form the B cell antigen receptor complex, were also expressed on the cell surface of epithelial cancer cells and co-located with IgM. Like the natural IgM, the epithelial cancer cell-derived IgM recognized a series of microbial antigens, such as single-stranded DNA, double-stranded DNA, lipopolysaccharide, and the HEp-2 cell antigen. More important, stimulation of the toll-like receptor 9 (TLR9), which mimics bacterial infection, substantially increased the secretion of IgM in human epithelial cancer cells. These findings indicate that human epithelial cancer cells as well as non-cancer epithelial cells can spontaneously produce IgM with natural antibody activity.

## Introduction

It is well known that as a classic immunity molecule, immunoglobulin (Ig) plays an essential role in immune system [1]. It can attach to foreign substances such as bacteria and assist in destroying them [2]. Ig was previously thought to be produced only by B lymphocytes and plasma cells. During the last decade, however, this concept has been challenged by a series of studies [3], [4], [5], [6], . In 2003, we first reported IgG expression in human epithelial cancer cells [3]. Since then, our group and others have confirmed that many human non-B cancer cells and some normal cells can produce Ig, especially IgG or IgA [4], [5], [6], [7], [8], [9], [10], [11], [12], [13], [14], [15], [16], [17]. Moreover, these non-B cancer cell-derived IgG or IgA is involved in the survival and proliferation of cancer cells [3], [4], [18]. However, the expression of IgM in human non-B cells is rarely studied [8]. Recently, we found that IgM heavy chain (Ig µ) gene with a distinct repertoire was transcribed in human epithelial cancer cells [8], suggesting that IgM might be also expressed in these epithelial lineage cells.

There are two classes of IgM, natural and immune. Natural IgM has been thought to be produced only by innate-like B-1 B cells in the absence of pathogen encounters, and immune IgM is produced by both innate-like B-1 B cells and adaptive B-2 B cells following an antigen or pathogen encounter. Natural IgM constitutes the majority of total circulating IgM. Most of the natural IgM is germline encoded and polyreactive, and it binds with low affinity to a number of different antigens, such as microbial pathogens, contributing to early immunity prior to the onset of the adaptive humoral response and playing a fundamental role in early antimicrobial immunity [19]. However, recent studies by Zhou et al. showed that not all polyreactive natural IgM-producing antigen-binding B cells express B-1 B cell surface markers (e.g., IgM^hi^, IgD^lo^, B220^lo^, Mac-1^hi^, CD23^lo^ and CD5^hi^) [20], suggesting that, besides B-1 B cells, other cell types may also be involved in the production of natural IgM.

Toll-like receptors (TLR) are a class of proteins that play a fundamental role in the innate immune system. They recognize “pathogen-associated molecular patterns”, which are structurally conserved molecules derived from microbes and are distinguishable from host molecules, and activate innate immune responses [21]. More than 13 members of the TLR family have been identified in mammals. TLR9 specifically recognizes unmethylated CpG sequences in microbial DNA [21], [22]. Synthetic oligodeoxynucleotides (ODN) with unmethylated CpG motifs, which can mimic the effects of microbial DNA, are also recognized by TLR9 [23], [24], [25], [26]. Once activated, TLR9 and its associated adapters, such as myeloid differentiation antigen 88 (MyD88) [27], [28], recruit intracellular signaling mediators and induce activation of the nuclear factor-κB (NF-κB) and mitogen-activated protein kinase pathways, resulting in the production of cytokines and Ig, mainly IgM [29].

In humans, TLR9 is expressed preferentially in B cells, plasmacytoid dendritic cells, monocytes, and natural killer cells [22]. Functional TLR9 have also been found in many human epithelial cancer cells such as lung cancer, breast cancer and prostate cancer [30], [31], [32], [33], [34]. More important, CpG ODN, and even non-CpG ODN, can activate TLR9 expressed in breast cancer cell lines and prostate cancer cell lines, resulting in increased cellular invasion [32], [33], [34]. When activated by unmethylated CpG, TLR9 induces the secretion of many cytokines, such as interleukin (IL)-1α, IL-6, and IL-8 [31], [35]. It is still unclear whether TLR9 on epithelial cancer cells can mediate IgM production and secretion.

In this study, we assessed IgM expression in human non-B cells, and showed that human epithelial cancer cells as well as non-cancer epithelial cells can spontaneously produce natural IgM. TLR9 agonists stimulated the secretion of IgM in human epithelial cancer cells. Like the B-1 B cell-derived natural IgM, the epithelial cancer cell-derived IgM recognized a series of microbial antigens and some autoantigen. These findings indicate that human epithelial cancer cells as well as non-cancer epithelial cells can spontaneously produce IgM with natural antibody activity.

## Materials and Methods

### Cell Lines and Culture

The human cervical cancer cell line HeLa MR, an *O* ^6^-methylguanine-DNA methyltransferase-deficient derivative of HeLa S3 [36], [37], [38], [39], [40], [41], was a gift from Dr. Shouping Ji (Academy of Military Medical Science, Beijing, China). All other cell lines, including human cervical cancer cell line HeLa, colon cancer cell lines HT-29 and SW480, hepatic cancer cell line HepG2, osteosarcoma cell line U-2 OS, embryonic kidney cell lines 293 and 293T, and B lymphocytic leukemia cell line Raji were obtained from American Type Culture Collection (Manassas, VA, USA). HeLa MR, HT-29, SW480, HepG2, U-2 OS, 293 and 293T were cultured in Dulbecco’s modified Eagle’s medium (DMEM) supplemented with 10% fetal bovine serum (FBS) and 1% antibiotics, while HeLa and Raji were cultured in RPMI 1640 supplemented with 10% FBS and 1% antibiotics at 37°C in a humidified atmosphere containing 5% CO_2_. The cells in logarithmic growth phase were used for the experiments.

### Tissue Samples

Tissue microarrays were purchased from the Department of Pathology, Beijing Friendship Hospital. We studied a total of 202 samples, which included 26 different tumor types, 19 counterpart samples of benign disease, and normal tissues. We cut 4-µm sections of the resulting multitumor tissue microarray blocks and mounted each section on an adhesive-coated slide (Instrumedics Inc., Hackensack, NJ, USA). Frozen cancer tissues, including breast cancer (2 cases), colon cancer (2 cases), lung cancer (2 cases), and ovarian cancer (1 case) were from the tumor tissue specimen bank of Peking University Cancer Hospital. 4-µm frozen sections were prepared and fixed with acetone.

### Immunohistochemistry

Tissue microarray sections were deparaffinized, rehydrated through ethanol washes of graduated concentrations, placed in 10 mM citrate buffer (pH 6.0), and then heated twice in a microwave oven for 5-minute each. The slides were then incubated with 3% hydrogen peroxide for 5 minutes, washed with phosphate-buffered saline (PBS), and blocked in PBS plus 10% normal goat serum for 10 minutes. After removing excess blocking buffer, we performed indirect immunohistochemical staining with monoclonal mouse anti-human Ig µ (1∶100, Dako, Carpinteria, CA, USA). Slides were incubated in a humidified chamber at 37°C for 45 minutes, washed thoroughly, and then incubated with goat anti-mouse IgG-horseradish peroxidase (HRP) (1∶100, Dako) at 37°C for 30 minutes. Slides were washed again, and bound antibodies were detected using 3,3′-diaminobenzidine tetrahydrochloride (DAB, Dako). Control sections were stained with goat anti-mouse IgG-HRP alone.

### Immunofluorescence Staining

To confirm the expression of IgM in human epithelial cells, two-color immunofluorescence staining was performed on frozen tissue slides. Briefly, the frozen tissue slides were blocked with 10% normal goat serum, and then the slides were incubated with mouse anti-human Ig µ (Mabworks Biotech Co., Beijing, China) as well as rabbit anti-human pan-cytokeratin or rabbit anti-human CD19 (Santa Cruz, CA, USA) at 37°C for 1 hour, followed by incubation with TRITC-conjugated goat anti-mouse IgG and Alexa Fluor® 488-conjugated goat anti-rabbit IgG (Santa Cruz) at 37°C for 30 minutes. After counterstaining with Hoechst 33342, the slides were observed directly under an inverted fluorescence microscopy (Olympus IX71-141, Tokyo, Japan).

### Flow Cytometry Analysis

We performed flow cytometry analysis to assess the expression of IgM and TLR9 in human epithelial cancer cells. For detection of IgM and TLR9 on cell surface, the cells were harvested, washed twice with PBS, blocked with 2% FBS in PBS at 4°C for 30 minutes, stained with mouse monoclonal antibodies (mAb) against human Ig µ (Mabworks) or TLR9 (IMG-305A, Imgenex, San Diego, CA, USA) at 4°C for 40 minutes. After two washes with PBS, the cells were incubated in 100 µl PBS containing fluorescein isothiocyanate (FITC)-conjugated goat anti-mouse IgG (Santa Cruz) at 4°C for 30 minutes. The cells were washed twice with PBS, and 10,000 cells were analyzed on a FACSCalibur flow cytometer using CellQuest software (Becton Dickinson, San Diego, CA, USA). Background fluorescence was determined using cells incubated with the secondary antibody but without the primary antibody.

For assessment of intracellular IgM and TLR9, the harvested cells were fixed in 4% paraformaldehyde at room temperature for 30 minutes, washed with PBS, and permeabilized twice with 1× permeabilization buffer (eBioscience, San Diego, CA, USA). The cells were then centrifuged at 1,500 rpm at 4°C for 5 minutes. The supernatant was discarded, and the cells were labeled with appropriate primary and secondary antibodies as described above.

To exclude the possibility of contamination of cancer cell lines by B lymphocytes, the cells were harvested, washed twice with PBS, blocked with 2% FBS in PBS for 30 minutes, and stained with monoclonal anti-human CD19-phycoerythrin (PE) (Becton Dickinson) at 4°C for 40 minutes. After two washes, the cells were assessed for CD19 expression on a FACSCalibur flow cytometer. The CD19^+^ cell line Raji was used as a positive control. Background fluorescence was determined using cells incubated with PE-conjugated mouse IgG (Becton Dickinson).

### Reverse Transcription-polymerase Chain Reaction (RT-PCR) and Realtime PCR Analyses

Total RNA was extracted from cells or tissue specimens using TRIzol reagent (Invitrogen, Carlsbad, CA, USA) and was treated with TURBO DNase (Ambion, Austin, TX, USA) to eliminate contamination of genomic DNA. Reverse transcription was performed with the RevertAid First Strand cDNA synthesis kit (Fermentas, Glen Burnie, MD, USA) according to the manufacturer’s instructions. The resulting cDNA was subjected to PCR and realtime PCR analyses.

PCR was performed to analyze the expression of Ig µ, Ig κ, CD79A, CD79B, TLR9 and MyD88 in the human epithelical cancer cell lines (primers shown in Table S1). The PCR products were separated by gel electrophoresis on 1% agarose. The identity of the PCR products was further confirmed by DNA sequencing.

Two-step realtime PCR was performed to quantify the expression of IgM and CD19 in the human epithelial cancer tissues using SYBR Green Master Mix (Applied Biosystems, Foster City, CA, USA) according to the manufacturer’s instructions (primers shown in Table S2). The reaction was run on the 7300 Fast Realtime PCR System (Applied Biosystems). Gene expression was quantified relatively to the expression of the housekeeping gene GAPDH, and normalized to the positive control (human peripheral blood mononuclear cells) by standard 2^−△△CT^ calculation.

### Protein Extraction and Western Blot Analysis

To extract cytoplasmic proteins, the harvested cell pellets were lysed in radioimmunoprecipitation assay (RIPA) lysis buffer (Thermo Fisher Scientific Inc., Rockford, IL, USA), and incubated on ice for 30 minutes. The lysates were centrifuged at 16,000 rpm at 4°C for 30 minutes. The supernatants were collected for Western blot analysis.

To obtain secreted proteins, the cell culture supernatant without cell debris was collected, precipitated overnight with 100% saturated ammonium sulfate (1∶1), and centrifuged at 16,000 rpm for 20 minutes at 4°C. The supernatant was discarded. The pellet was resuspended in 100 µl PBS, dialyzed twice with 0.05 M PBS for 24h, and then used for Western blot analysis.

Western blot analysis was performed using reduced (with β-mercaptoethanol) or non-reduced (without β-mercaptoethanol) protein samples that were separated by SDS-polyacrylamide gel electrophoresis (PAGE) and transferred onto nitrocellulose membranes (Amersham Pharmacia, Little Chalfont, UK). The membranes were blocked with Tris-buffered saline containing 0.1% Tween-20 and 5% nonfat milk or 5% bovine serum albumin (for detecting phosphoproteins) at room temperature for 2 hours, and were incubated with primary antibodies, such as mouse anti-human Ig µ mAb (Mabworks), goat anti-human Ig µ polyclonal antibody (Sigma, St. Louis, MO, USA), mouse anti-human CD79A and CD79B mAbs (Abcam, Cambridge, MA, USA), mouse anti-human TLR9 mAb (Imgenex), anti-phospho-PKC mAb, anti-phospho-Akt mAb, anti-phospho-ERK mAb, anti-phospho-IκB mAb, and anti-IκB mAb (all from Cell Signaling, Danvers, MA, USA), rabbit anti-ERK polyclonal antibody (KangCheng Biotech Co., Shanghai, China), and anti-actin mAb (Sigma), at 4°C overnight. The membranes were washed three times with Tris-buffered saline containing 0.1% Tween-20 for 10 minutes each, and incubated with IRDye 800− or 700-conjugated secondary antibodies (LI-COR Bioscience Inc., Lincoln, NE, USA) at room temperature in the dark for 1 hour. The signal was detected using the Odyssey Imaging System (LI-COR Bioscience).

### Intracellular Calcium Flux Measurement

Measurement of intracellular calcium flux was performed as described previously [42]. Briefly, HeLa MR cells were grown in specialized glass-bottom microwell dishes (MatTek Corp., Ashland, MA, USA) and loaded with 5 µM Fluo-3/AM in HEPES-buffered saline at 37°C in the dark for 30 minutes. The cells were washed with HEPES-buffered saline and stimulated with 20 µg/ml goat anti-human IgM or goat IgG. Fluorescence was monitored at 488 nm (excitation) and 530 nm (emission) using a Leica TCS-NT confocal fluorescence microscope (Leica MicroImaging, Mannheim, Germany) with a 40× oil immersion lens (Wetzler, Heidelberg, Germany). Images were collected at 5 seconds for five times before the stimulation, and every 1.5 seconds for 60 seconds, then every 5 seconds for another 120 seconds. The measurement was completed at room temperature, and each field of cells was selected randomly. The images were analyzed for relative fluorescence using Leica confocal software (Wetzler). All calcium flux assays were performed in the presence of extracellular calcium and in the absence of EDTA or EGTA in the assay buffers. Therefore, both intracellular calcium release and extracellular calcium influx were analyzed.

### Cell Treatment with ODN Stimulation

HeLa MR cells were cultured in DMEM supplemented with 10% FBS before ODN stimulation. The medium was then changed to DMEM supplemented with 2% FBS, and 10 µg/ml of a stimulus, such as phosphorothioate-modified, human-specific CpG 2006 [5′-TCGTCGTTTTGTCGTTTTGTCGTT-3′], non-CpG ODN controls CpG 2078 [5′-TGCTGCTTCCCCCCCCCCCC-3′] and GpC [5′-TGCTGCTTTTG TGCTTTTGTGC TT-3′], or neutralizing CpG-N ODN208 [5′-TGCCGCGGCAGA-3′]) was added with or without 10 µmol/l chloroquine (Sigma) for 1 hour of preincubation before ODN was added. After 3 days, the cells were harvested for RT-PCR, flow cytometry, and Western blot analysis. The cell culture supernatant also was collected for Western blot as described above.

MyD88 knockdown assay was performed using synthesized MyD88 siRNAs as described previously [43]. The siRNA was transfected into HeLa MR cell line by electroporation. The above-mentioned stimulus (CpG 2006, CpG 2078, or GpC) was added into the cell culture 24 hours after elecroporation, and the cells were harvested after another 3 days for RT-PCR, flow cytometry, and Western blot analyses.

### Confocal Microscopy Analysis

HeLa MR cells were stimulated with or without goat anti-human Ig µ polyclonal antibody (20 µg/ml) for 5 minutes, and double stained with PerCP/Cy5.5-conjugated mouse anti-human Ig µ (Biolegend, San Diego, CA, USA) and FITC-conjugated mouse anti-human CD79A (AbD SEROTEC, Kidlington, UK) or FITC-conjugated mouse anti-human Ig µ and PE-conjugated mouse anti-human CD79B (eBioscience). The cells then underwent confocal microscopy analysis, and the images were collected with an Axioskop-2 microscope (Olympus, Tokyo, Japan), a 63× NA 0.75 Plan Apochromat oil immersion objective and standard filter sets (Leica MicroImaging), a 1300×1030 pixel-cooled charge-coupled device camera (CCD-1300-Y; Princeton Instruments, Trenton, NJ, USA), and Metavue software (Visitron Systems, Puchheim, Germany).

### Analysis of Natural Antibody Activity of Epithelial Cancer Cell-derived IgM

To analyze whether epithelial cancer cell-derived IgM has natural antibody activity against the microbial antigens such as single-stranded DNA (ssDNA), double-stranded DNA (dsDNA), or lipopolysaccharide (LPS), we performed antigen-specific enzyme-linked immunosorbent assay (ELISA) using HeLa MR cell culture supernatant containing IgM. Microtiter plates were coated with 10 µg/ml ssDNA, dsDNA, or LPS (all from Sigma). Mouse anti-human Ig µ mAb and HRP-labeled goat anti-mouse IgG were used to detect IgM, with tetramethylbenzidine as the substrate. OD450 was measured using a microplate reader (Bio-Tek, Winooski, VT, USA).

We also performed indirect immunofluorescence staining to analyze autoantibody activity of epithelial cancer cell-derived IgM. We incubated HEp-2 cell slides (EUROIMMUN, Luebeck, Germany) with HeLa MR cell culture supernatant containing IgM. After washing with PBS, mouse anti-human Ig µ mAb and FITC-conjugated goat anti-mouse IgG were used to detect positive signal.

### Statistical Analysis

All statistical analyses were performed with the statistical software SAS version 8.1 (SAS Institute Inc., Cary, NC, USA). Differences between various groups were calculated by Student’s *t* test or chi-square test. Differences were considered statistically significant when *P* was <0.05.

## Results

### IgM is Mainly Expressed in Human Epithelial Cells

**Figure 1:**
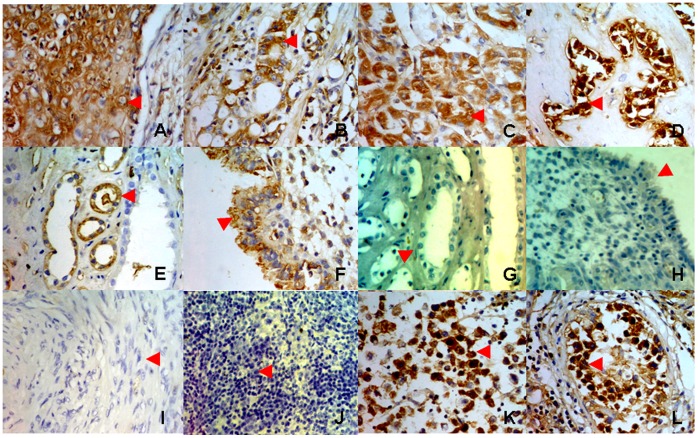
Detection of IgM expression by immunohistochemistry on tissue microarray. A, lung cancer cells; B, breast cancer cells; C, liver cancer cells; D, pancreatic cancer cells; E, renal tubule epithelial cells; F, endometrium epithelial cells; G, renal tubule epithelial cells, stained with goat anti-mouse IgG-HRP only, as a negative control; H, endometrium epithelial cells, stained with goat anti-mouse IgG-HRP only, as a negative control; I, leiomyoma cells; J, T lymphoma cells; K, seminoma cells; L, spermatocytes.

We analyzed tissue microarray of 202 tissue samples for IgM expression by immunohistochemistry, including cancer or normal tissues of epithelial, mesenchymal, and neuroglial origin as well as germ cells (Table S3). We found that IgM was expressed more frequently in epithelial cells, including 17 of 34 (50%) epithelial cancer cells, especially carcinomas of the lung, breast, liver, and pancreas (Figure 1A–D), and 23 of 66 (34.8%) non-cancer epithelial cells (Figure 1E, F). In contrast, no or low-frequency IgM was found in neoplastic mesenchymal cells (1 of 47, [2.1%]; including lipoma, fibroma, and leiomyoma cells [Figure 1I]) and in normal mesenchymal cells (1 of 34, [2.9%]; including lipocytes, fibroblasts, and smooth muscle cells). None of the neuroglial cells assessed expressed IgM (0 of 13, [0%]). It is interesting that IgM expression was detected at high levels in seminoma cells and in some normal germ cells in the testis (Figure 1K, L). Two cases of T cell lymphoma had no detectable IgM expression, as expected (Figure 1J). These results suggest that non-B cell-derived IgM is found mainly in epithelial cells.

**Figure 2:**
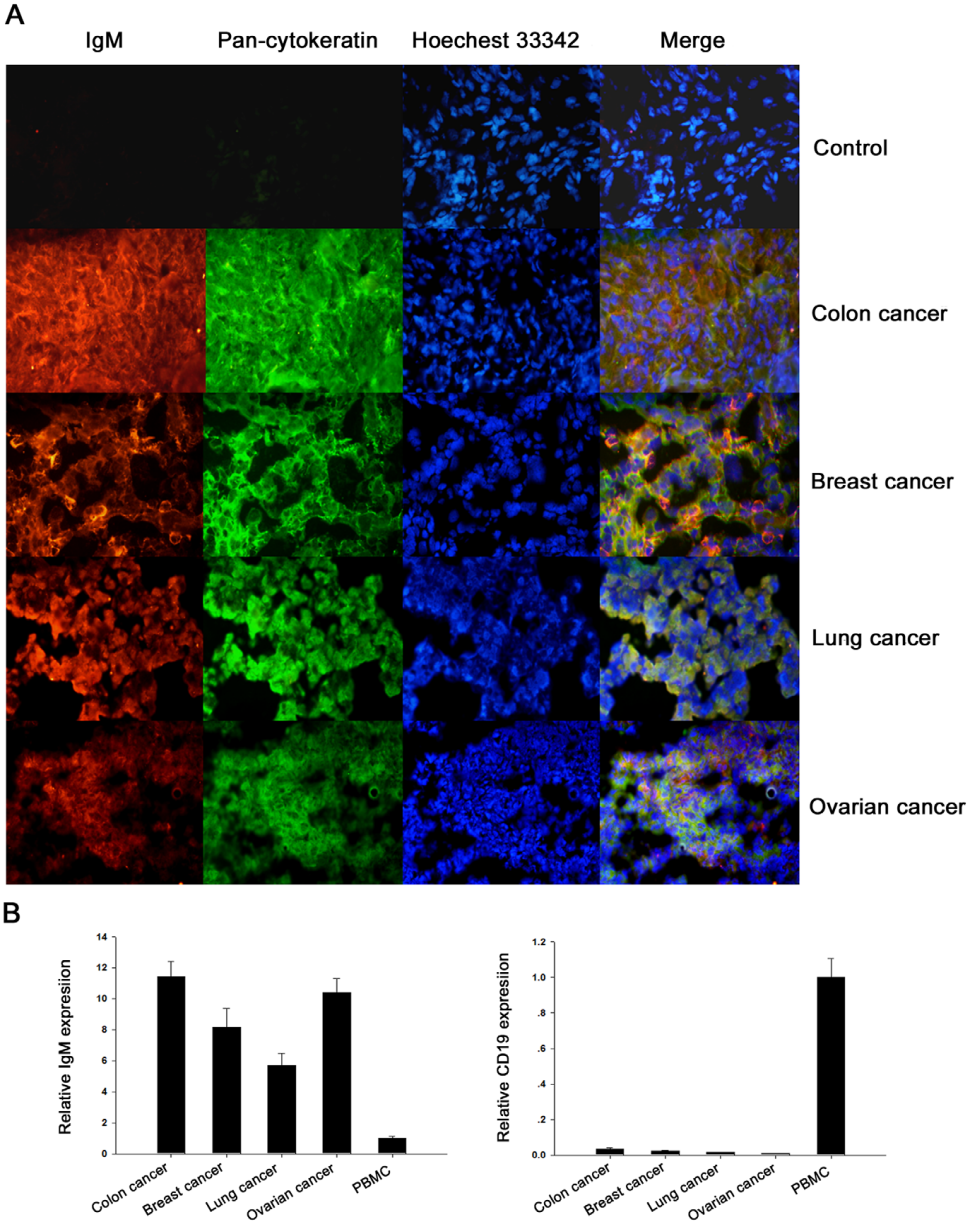
Epithelial cells are the main producers of IgM in the epithelial cancer tissues. A, co-localization in human epithelial cancers of IgM and cytokeratin. Human epithelial cancers, including colon cancer, breast cancer, lung cancer, and ovarian cancer, were double stained with anti-human IgM (red), anti-human pan-cytokeratin (green), and Hoechest 33342 (blue). The colocalization of IgM and cytokeratin was shown (yellow). Colon cancer stained with TRITC-conjugated goat anti-mouse IgG and Alexa Fluor® 488-conjugated goat anti-rabbit IgG only was used as a negative control. B, there was no correlation between the levels of IgM transcripts and B-cell specific CD19 transcripts in human epithelial cancers. Human epithelial cancers, including colon cancer, breast cancer, lung cancer, and ovarian cancer were subjected to qRT-PCR analysis of IgM and CD19 expression. Human peripheral blood mononuclear cells (PBMC) were used as the positive control for IgM and CD19 expression.

To further confirm that the IgM is expressed by the epithelial cancer cells but not the infiltrated B cells, two-color immunofluorescence staining was performed on frozen epithelial cancer tissue slides, including colon cancer, breast cancer, lung cancer, and ovarian cancer, with anti-IgM and anti-pan-cytokeratin antibodies. As shown in Figure 2A, a significant co-localization was revealed bewteen the IgM and cytokeratin; only few B cell infiltration was detected in these tissues as demonstrated by IgM and CD19 double staining (Figure S1). Moreover, realtime PCR analysis demonstrated that there was no correlation between the levels of IgM transcripts and B-cell specific transcripts CD19 in these tissues, as they expressed higher levels of IgM than human peripheral blood mononuclear cells (PBMC), but expressed pretty lower levels of CD19 than PBMC (Figure 2B). All these results suggest that the IgM positive signal was derived from epithelial cancer cells.

### IgM is Widely Expressed in Several Human Epithelial Cancer Cell Lines

**Figure 3:**
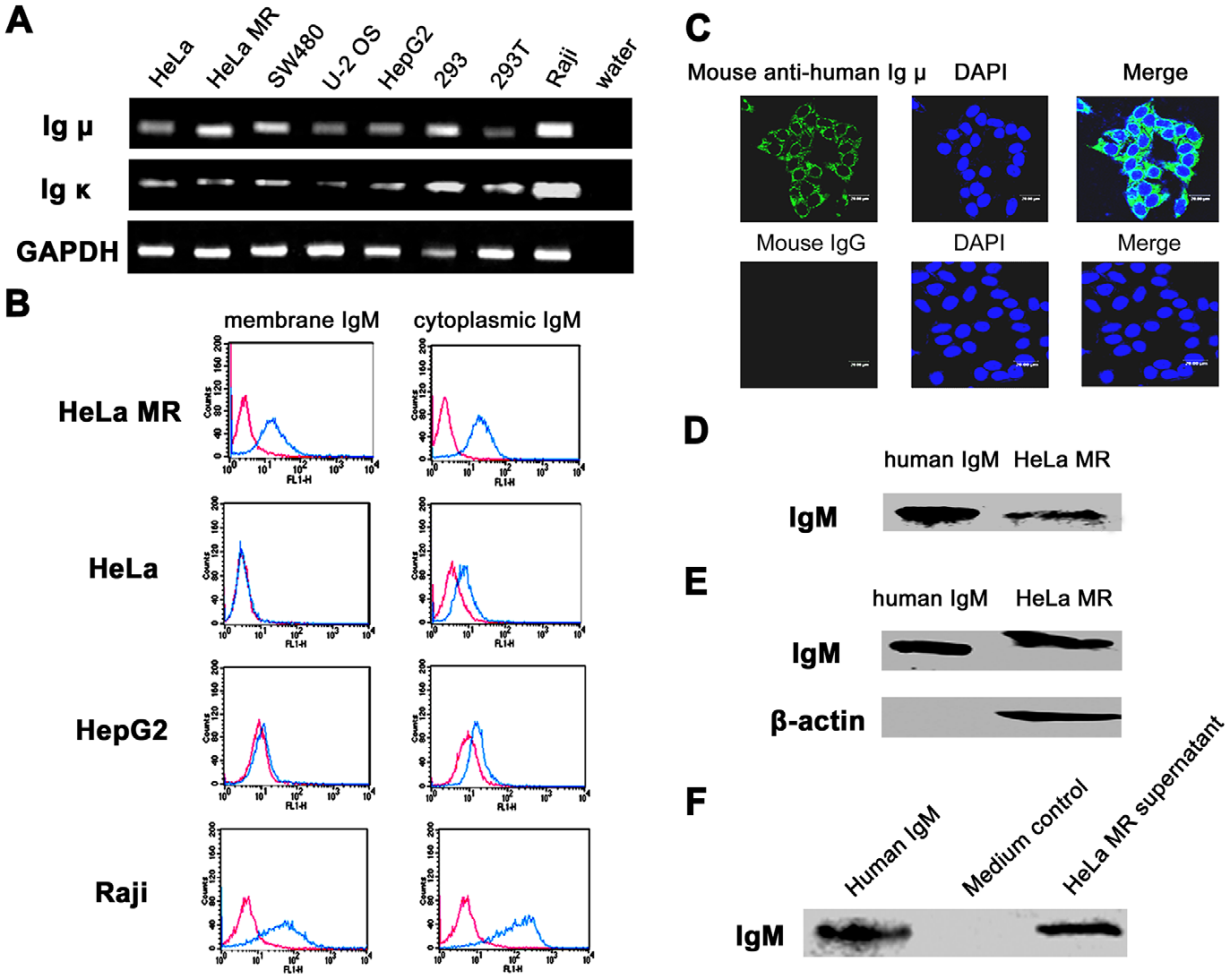
IgM expression in human non-B cell-derived cancer cell lines. A, detection of Ig µ and Ig κ transcripts in multiple cancer cell lines by semi-nested RT-PCR. HeLa and HeLa MR, cervical cancer cell lines; SW480, colon cancer cell line; U-2 OS, osteosarcoma cell line; HepG2, hepatic cancer cell line; 293 and 293T, human embryonic kidney cell lines. Raji, human B lymphocytic leukemia cell line, as positive control; GAPDH, internal control. B, flow cytometry study using mouse anti-human Ig µ mAb showed that IgM was localized not only on the plasma membrane but also in the cytoplasm of epithelial cancer cells, especially HeLa MR cells. Red line, isotype control IgG1; Blue line, anti-human IgM. C, Confocal microscopy analysis of HeLa MR cells using mouse anti-human Ig µ mAb showed that IgM was present both on the cell membrane and in the cytoplasm. Mouse IgG, as isotype control. D, whole IgM was detected in HeLa MR cells by non-reducing SDS-PAGE (without β-mercaptoethanol) and Western blot with goat anti-human Ig µ polyclonal antibody. Human IgM, as positive control. E, IgM expression was detected in HeLa MR cells by reducing SDS-PAGE (with β-mercaptoethanol) and Western blot with mouse anti-human Ig µ mAb. Human IgM, as positive control; β-actin, internal control. F, IgM was also detected in the cultural supernatant of HeLa MR cells by reducing SDS-PAGE and Western blot using mouse anti-human Ig µ mAb. Human IgM, as positive control; Medium, as negative control.

We previously found the expression of monoclonal, rearranged Ig µ in HeLa S3 and HT-29 cells [8]. To determine whether IgM was widely expressed in non-B cells, especially epithelial cancer cell lines, we assessed the expression of Ig µ and Ig κ genes in four human epithelial cancer cell lines (cervical cancer cell lines HeLa and HeLa MR, colon cancer cell line SW480, and hepatic cancer cell line HepG2), one osteosarcoma cell line (U-2 OS), and two human embryonic kidney cell lines (293 and 293T) by RT-PCR. Raji cells were used as a positive control. The results revealed that Ig µ and Ig κ transcripts were detected in all these cell lines (GenBank accession No. FJ197674, Figure 3A). Sequence analysis revealed that the Ig κ had a predominant Vκ4-1/Jκ3 recombination pattern (data not shown).

Flow cytometry analysis revealed membranous and cytoplasmic expression of IgM in HeLa MR cells, and little or no expression of membranous IgM with low expression of cytoplasmic IgM in HeLa and HepG2 cells (Figure 3B). Then the HeLa MR was chosen as a cell model for further analysis. Confocal microscopy confirmed the localization of IgM to be on the membrane and in the cytoplasm (Figure 3C). Reducing and non-reducing Western blot analysis revealed the expression of IgM in the cells as well as the cell culture supernatant (Figure 3D–F).

As one of the most important controls, we found that none of the cancer cell lines assessed expressed surface CD19 except Raji cell which was used as a positive control (Figure S2). These findings exclude the possibility of contamination of cancer cell lines by B lymphocytes.

### Expression of BCR-like Complex in Human Epithelial Cancer Cells

**Figure 4:**
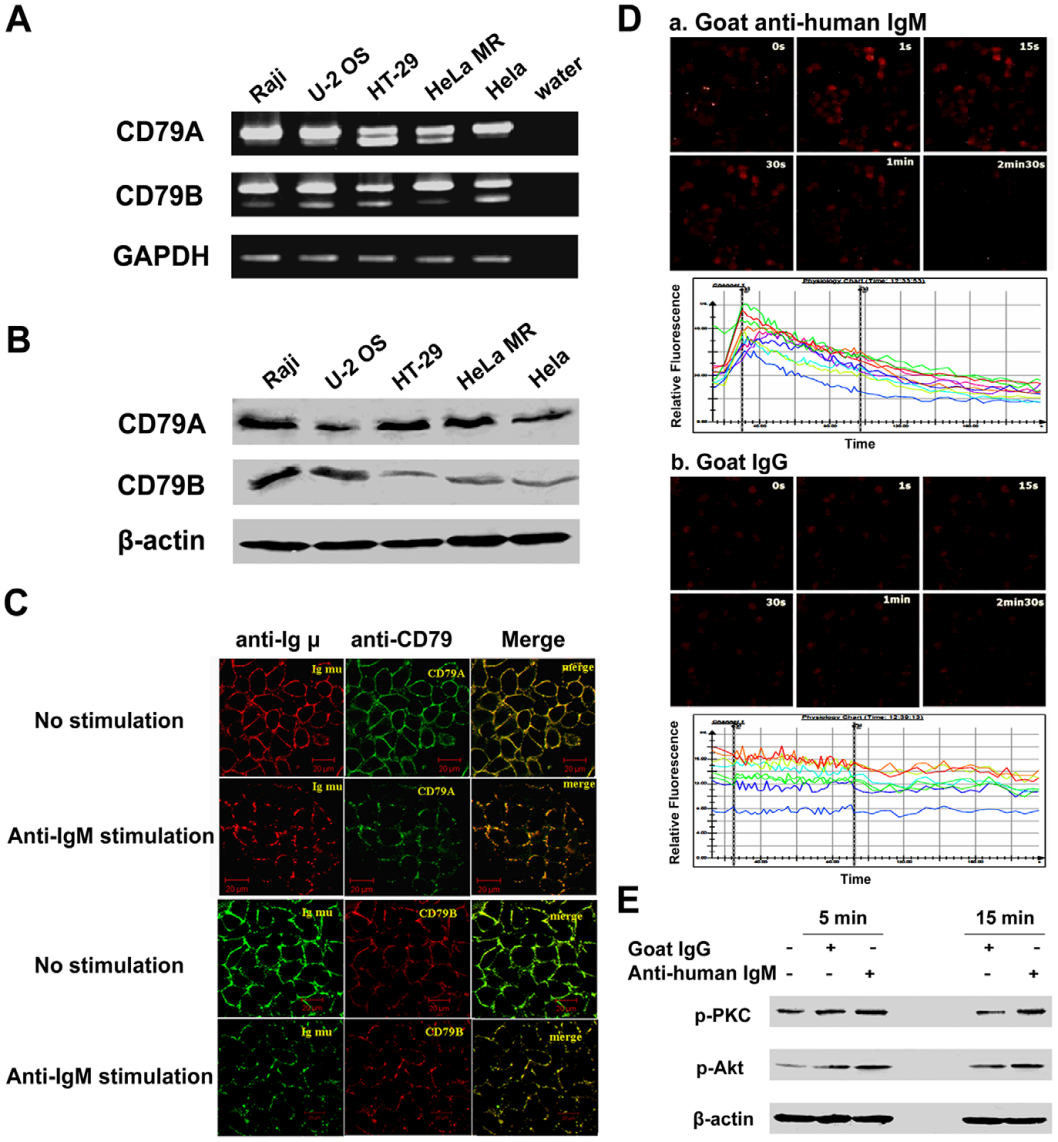
Expression of BCR-like complex in human epithelial cancer cells. A, RT-PCR showed that CD79A and CD79B were detected in human cancer cell lines, U-2 OS, HT-29, HeLa MR and HeLa, and that there were two isoforms for both CD79A and CD79B. Raji, as positive control; GAPDH, internal control. The PCR products were separated by gel electrophoresis on 1% agarose and were further confirmed by DNA sequencing. B, Western blot analysis demonstrated the presence of the larger isoform of CD79A and CD79B. β-actin, internal control. C, confocal microscopy analysis demonstrated co-localization (yellow) of CD79A (FITC, green) and Ig M (PerCP/Cy5.5, red) (upper two panels), or CD79B (PE, red) and Ig M (FITC, green) (lower two panels) in HeLa MR cells with or without stimulation with anti-IgM. D, calcium flux analysis by confocal microscopy in HeLa MR cells loaded with Fluo-3/AM and stimulated with goat anti-human IgM (a) or goat IgG as isotype controle (b). Images were captured after stimulation for 0, 1, 15, 30, 60, and 150 seconds. The corresponding time course of the calcium flux is shown. The relative fluorescence from the images was estimated with Leica confocal software. Each line represents the signal derived from a single cell. E, phosphorylation of PKC and Akt downstream of the PLC-γ2 and PI3K signaling pathways, respectively, was detected in HeLa MR cells after stimulation with 20 µg/ml goat anti-human IgM for 5 and 15 minutes, respectively.

CD79A and CD79B, two B cell specific molecules, are physically linked to membranous IgM on the surface of B cells, forming the B cell antigen receptor (BCR) complex. To address whether CD79A and CD79B also are linked to epithelial cancer cell-derived IgM, forming a BCR-like complex, we first analyzed if these molecules could be expressed by these cells. By RT-PCR and Western blot analyses, we demonstrated the expression of CD79A and CD79B in epithelial cancer cells (Figure 4A, B). Using HeLa MR as a cell model, we further confirmed that these molecules were co-localized with the membranous IgM (Figure 4C). More important, it was noted that before stimulation, the BCR-like complex was uniformly distributed on epithelial cancer cells. After stimulation, anti-IgM antibody cross-linked the BCR-like complex into small and large patches (Figure 4C) and induced an increase in calcium flow (Figure 4D). Moreover, the BCR downstream signaling pathways, phosphatidylinositol 3-kinase (PI3K)-Akt and phospholipase C (PLC)-γ2-PKC, were activated after stimulation with anti-human IgM (Figure 4E). Taken together, these results suggest that CD79A and CD79B are linked to membranous IgM on the surface of epithelial cancer cells, forming a BCR-like complex, which may mediate signals leading to growth and survival of the epithelial cancer cells.

### Analysis of Natural Antibody Activity of Human Epithelial Cancer Cell-secreted IgM

**Figure 5:**
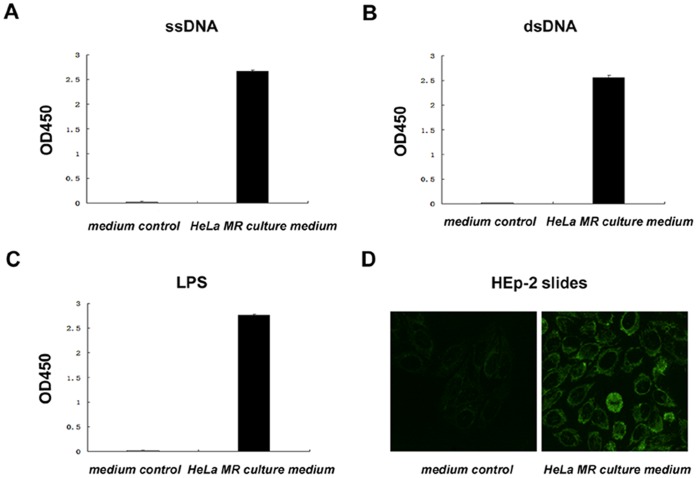
Human epithelial cancer cell-derived IgM recognized various self and non-self antigens. A–C, ELISA showed that secreted IgM in culture supernatant of HeLa MR cells could recognize ssDNA (A), dsDNA (B), and LPS (C). D, indirect immunofluorescence analysis showed that secreted IgM in culture supernatant of HeLa MR cells could recognize autoantigens in HEp-2 cells.

The spontaneous expression of IgM in the human epithelial cancer cells without evidence of infection or other stimulation urged us to detect whether they have the activity of natural IgM. For this purpose, we tested the ability of HeLa MR cell-derived IgM to recognize ssDNA, dsDNA, and LPS. By ELISA, we discovered that HeLa MR cell-secreted IgM recognized all of these three antigens (Figure 5A–C). In addition, this IgM had autoantibody activity and recognized the well-defined autoantigens in the nucleus, cytoplasm, and cytoskeleton of HEp-2 cells (Figure 5D).

### IgM Secretion by Human Epithelial Cancer Cells Upon TLR9 Agonist Stimulation

**Figure 6:**
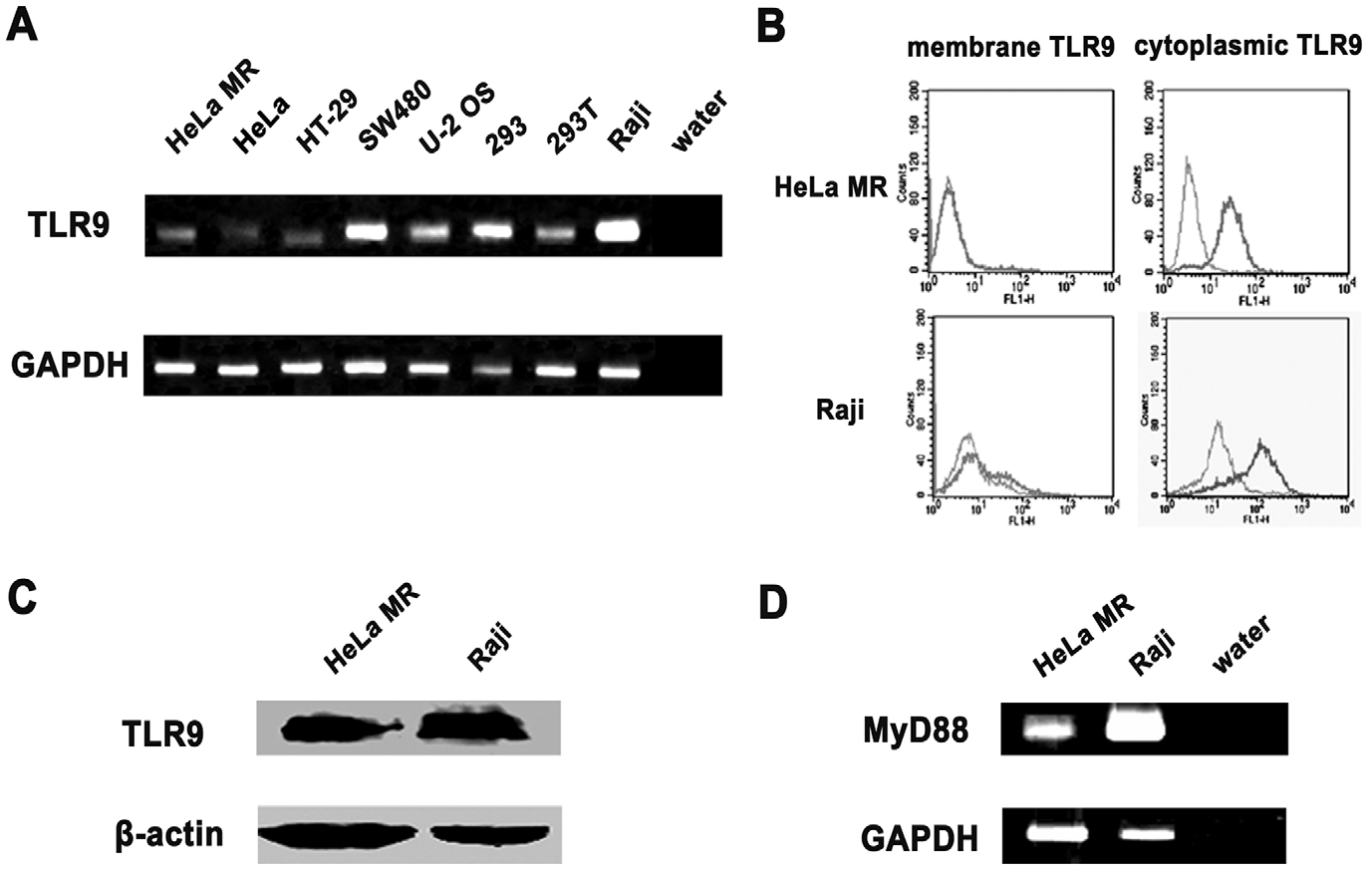
TLR9 expression was detected in non-B cancer cell lines. A, TLR9 mRNA was detected in several cancer cell lines, HeLa MR, HeLa, HT-29, SW480, U-2 OS, 293 and 293T, by RT-PCR. Raji, as positive control; GAPDH, internal control. B, TLR9 expression was detected in HeLa MR cells by flow cytometry. Gray, isotype control IgG1; Black, anti-human TLR9. C, TLR9 expression was detected in HeLa MR cells by Western blot. Raji, as positive control; β-actin, internal control. D, expression of the TLR9-associated adapter MyD88 was detected in HeLa MR cells by RT-PCR. Raji, as positive control.

**Figure 7:**
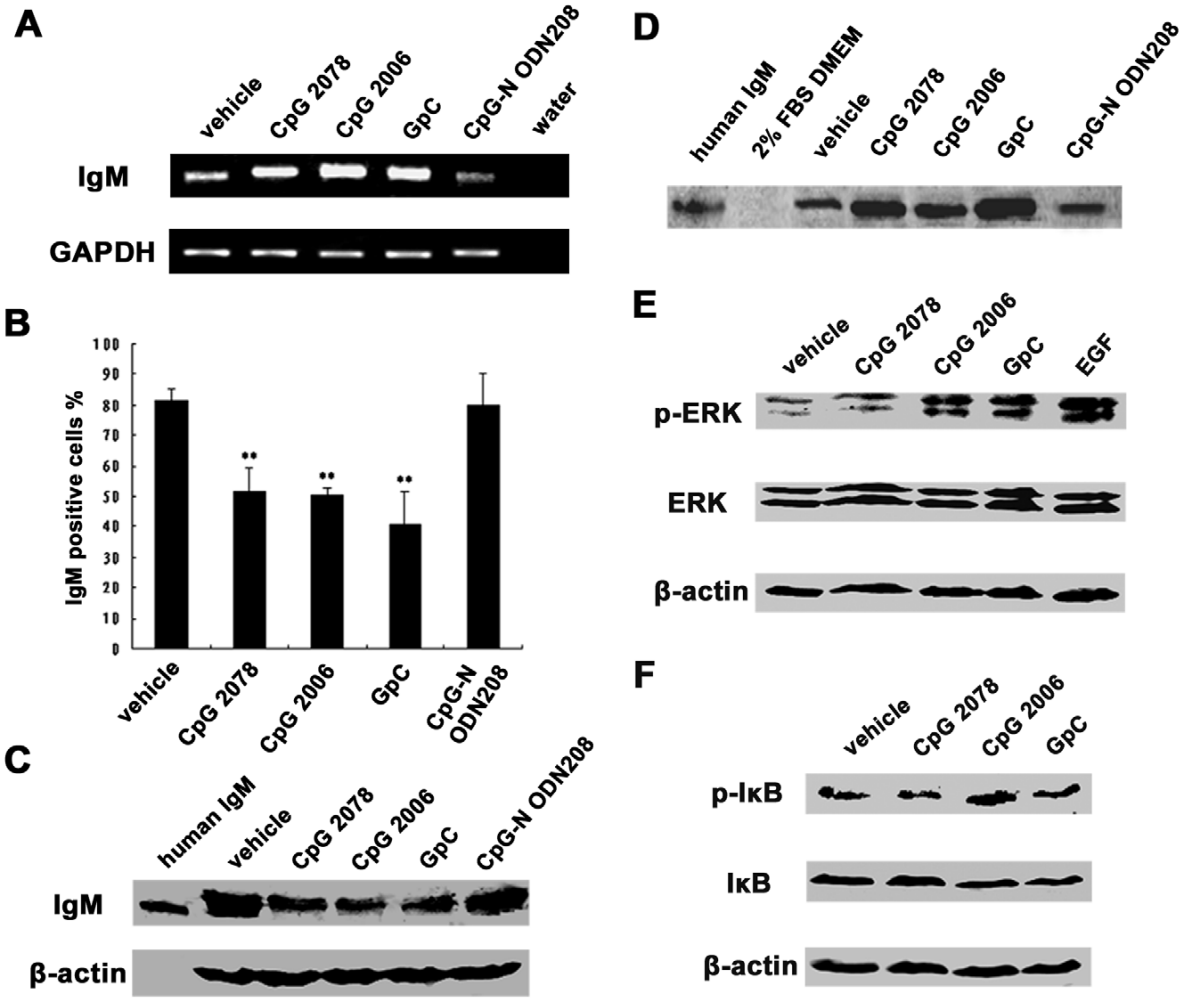
TLR9 agonists stimulated human epithelial cancer cells to secrete IgM. A, Ig µ mRNA level was analyzed by semiquantitative RT-PCR after stimulation by CpG 2006, and the two non-CpG ODN controls, CpG 2078 and GpC. CpG-N ODN208, as negative control; GAPDH, internal control. B, flow cytometry analysis showed that cytoplasmic IgM was decreased after stimulation with CpG 2006, CpG 2078, and GpC. CpG-N ODN208, as negative control. **P<0.01 *vs.* vehicle control. C, Western blot analysis showed that cytoplasmic IgM was decreased after stimulation with CpG 2006, CpG 2078, and GpC. CpG-N ODN208, as negative control; β-actin, internal control. D, Western blot analysis showed increased level of secreted IgM after stimulation with CpG 2006, CpG 2078, and GpC. CpG-N ODN208, as negative control. E, phosphorylation of ERK, a molecule downstream of TLR9 signaling cascade, was detected by Western blot after stimulation with CpG 2006, CpG 2078, and GpC. EGF, epidermal growth factor was used as a positive control for ERK phosphorylation. F, Western blot analysis showed that IκB, a molecule in NF-κB pathway, also was phosphorylated after stimulation with CpG 2006, CpG, 2078 and GpC.

We examined the expression of TLR9 in epithelial cancer cell lines, and found that TLR9 was expressed and located exclusively in the cytoplasm of epithelial cancer cells, as seen in B cells (Figure 6A–C). MyD88, a cytosolic adapter protein that has been reported to be an essential mediator for TLR9 signaling, was also expressed in these cells (Figure 6D). We further stimulated HeLa MR cells with type B CpG ODN CpG 2006, a molecule known to induce the secretion of especially IgM in B cells, and its two non-CpG ODN controls, CpG 2078 and GpC, as well as the neutralizing CpG-N ODN208. As expected in B cells, IgM expression was significantly upregulated after stimulation with CpG 2006 as shown by semiquantitative RT-PCR (Figure 7A). More important, CpG 2006 decreased the cytoplasmic IgM level substantially (Figure 7B, C), but the IgM level was increased in the supernatant (Figure 7D). We were surprised that the two non-CpG ODN controls, CpG 2078 and GpC, which have been considered to be unstimulatory controls for the TLR9 agonistic CpG 2006 in B cells, also increased IgM expression and secretion, with effects that were similar to or even stronger than the effects of CpG 2006 (Figure 7A–D), suggesting that the two non-CpG ODN controls also may have the ability to activate TLR9. As expected, CpG-N ODN208, which has been reported to be unable to activate TLR9 and, in fact, to inhibit the TLR9 pathway [44], had no effect on IgM secretion (Figure 7A–D). Moreover, stimulation by CpG 2006, CpG 2078, and GpC (but not CpG-N ODN208) substantially activated the ERK and NF-κB pathways, which are downstream of the TLR9 signaling cascade (Figure 7E, F).

**Figure 8:**
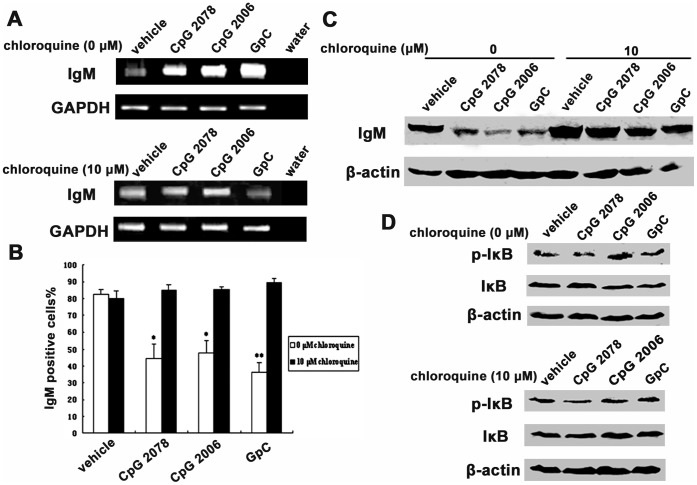
Chloroquine abolished effects for TLR9 agonists on IgM secretion in HeLa MR cells. A, semi-quantative RT-PCR showed increased Ig µ expression after stimulation by CpG 2006, CpG 2078 and GpC (upper panel). The effects of TLR9 agonists were abolished by chloroquine (lower panel). GAPDH, internal control. B, flow cytometry analysis showed that the cytoplasmic IgM level was decreased after stimulation with CpG 2006, CpG 2078 and GpC, and that this was abolished by chloroquine. *P<0.05 *vs.* vehicle control; **P<0.01 *vs.* vehicle control. C, Western blot analysis showed that the cytoplasmic IgM level was decreased after stimulation with CpG 2006, CpG 2078 and GpC, and that this was abolished by chloroquine. D, Western blot analysis showed that IκB was phosphorylated after stimulation with CpG 2006, CpG 2078 and GpC, and that this was abolished by chloroquine.

**Figure 9:**
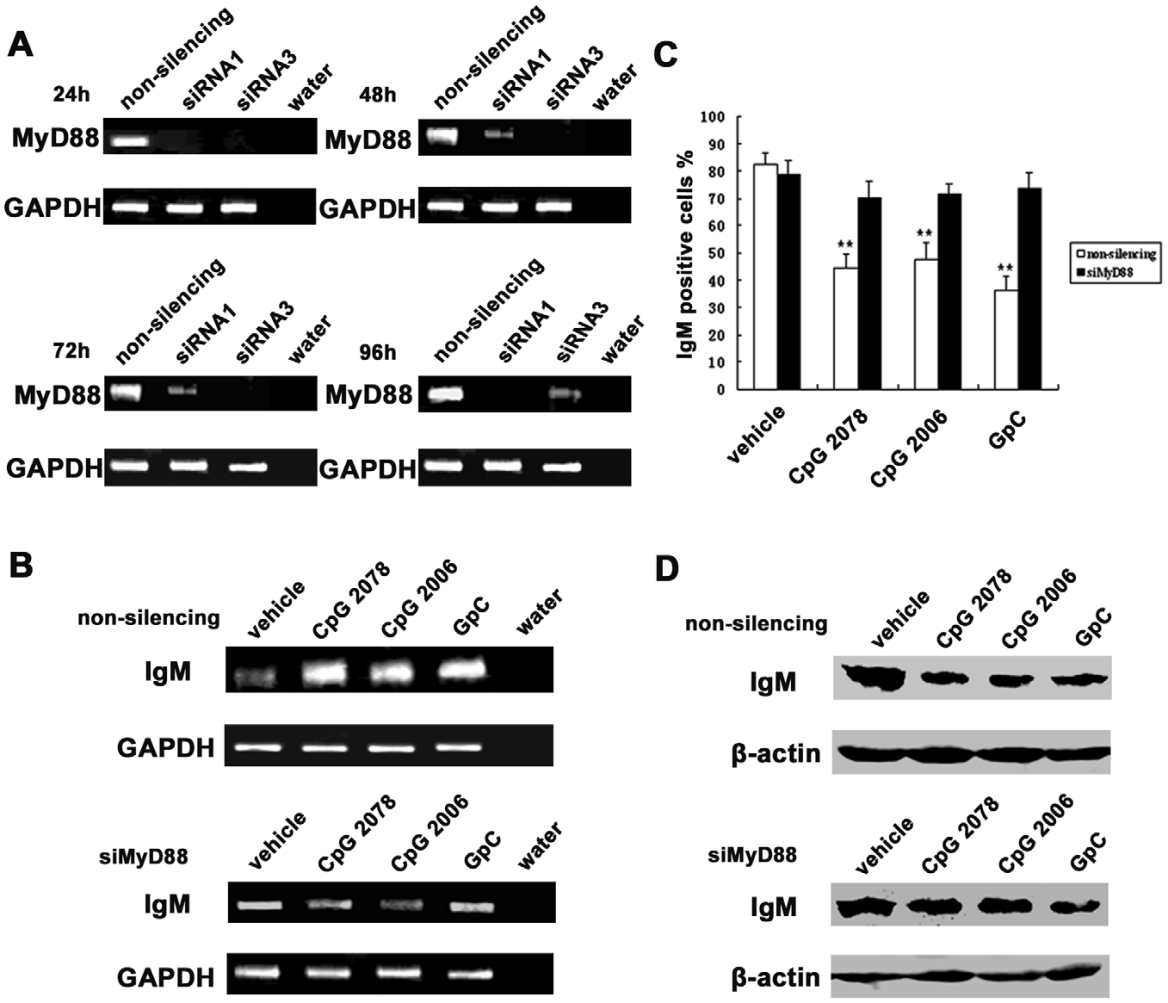
Knockdown of MyD88 by siRNA inhibited TLR9 agonist-induced IgM secretion in HeLa MR cells. A, effects of three synthesized siRNAs for knocking down MyD88 expression were analyzed after transfection from 24 h to 96 h by semiquantitative RT-PCR. Results of the effective siRNAs 1 and 3 are shown. B, semi-quantative RT-PCR showed increased Ig µ expression after stimulation by CpG 2006, CpG 2078 and GpC (upper panel). This was abolished by MyD88 knockdown with siRNA1 and siRNA3 (lower panel). GAPDH, internal control. C, flow cytometry analysis showed that the cytoplasmic IgM level was decreased after stimulation with CpG 2006, CpG 2078 and GpC, and that this was abolished by MyD88 knockdown with siRNA1 and siRNA3. **P<0.01 *vs.* vehicle control. D, Western blot analysis showed that the cytoplasmic IgM level was decreased after stimulation with CpG 2006, CpG 2078 and GpC, and that this was abolished by MyD88 knockdown with siRNA1 and siRNA3.

We further found that the addition of chloroquine (10 µmol/l), an inhibitor of endosomal maturation, inhibited CpG 2006-, CpG 2078-, and GpC-induced upregulation of the Ig µ mRNA level (Figure 8A). The decreasing trend of cytoplasmic IgM was also abolished after chloroquine treatment as shown by flow cytometry and Western blot analyses (Figure 8B, C). Moreover, the NF-κB pathway, which plays a essential role in Ig synthesis and secretion, was activated after stimulation by the TLR9 agonists and was deactivated by chloroquine (Figure 8D). Similarly, knocking down MyD88 by siRNA (Figure 9A) inhibited CpG 2006-, CpG 2078-, and GpC-induced upregulation and secretion of IgM (Figure 9B-D). Taken together, these results prove that TLR9 agonists can stimulate human epithelial cancer cells to increase the secretion of IgM via the TLR9-MyD88 pathway.

## Discussion

In this study, we further confirmed the new concept that Ig can be spontaneously produced from some non-B cells, especially human epithelial cancer cells. Similar to B cell-derived IgM, human epithelial cancer cell-derived IgM was not only expressed on the cell surface and co-localized with CD79 to form a BCR-like complex but also spontaneously secreted into supernatant. More interestingly, similar to B-1 B cell-derived natural IgM, epithelial cancer cell-secreted IgM showed significant natural antibody activity, and this activity was regulated by the TLR9-MyD88 pathway.

During the past several years, we and other groups have found that Ig, especially IgG and IgA, is frequently expressed in many non-B cells, including both epithelial and non-epithelial cells [3], [4], [5], [6], [7], [8], [9], [10], [11], [12], [13], [14], [15], [16], [17], [18]. Moreover, it has shown that human non-B cancer cell-derived IgG and IgA are involved in the survival and proliferation of cancer cells [3], [4], [18]. The expression of µ chain transcripts with a distinct repertoire also has been detected in some epithelial cancer cells [8]. In this study, using immunohistochemistry performed on tissue microarrays, we first analyzed the expression profile of IgM in different types of non-B cells, and found that IgM was preferentially expressed in many human epithelial cells, including normal and neoplasitc epithelial cells, as well as spermatocytes, but only rarely in mesenchymal cells.

Furthermore, we discovered that the epithelial cancer cell-derived IgM transcripts showed germline sequence; restricted usage patterns and even identical VHDJH rearrangement was frequently revealed among different types of epithelial cancer cells [8]. Notably, some epithelial cancer cell lines, such as HeLa S3 and HeLa MR, could simultaneously express IgG and IgM. Nevertheless, unlike B cells, there was no identical pattern between VH_γ_D_γ_JH_γ_ and VH_µ_D_µ_JH_µ_ in these epithelial cancer cells, which needs to be further studied.

IgM can be secreted from B cells as a pentamer, but it is also expressed as membrane-bound antibody on all naive B cells [45]. Membranous IgM binds with CD79 to form the BCR complex. The BCR is an integral membrane protein complex composed of two Ig heavy chains, two Ig light chains, and two heterodimers of CD79A (Ig-α) and CD79B (Ig-β). After initiation of BCR ligation by antigen (or anti-IgM antibody used as surrogate for antigen), the BCR is cross-linked and the three main protein tyrosine kinases, the SRC family kinases LYN and SYK and the TEC family kinase BTK, are activated. PI3K and PLC-γ2 are important downstream effectors of BCR signaling. This signaling ultimately results in the expression of immediate early genes that further activate the expression of other genes involved in B cell proliferation, differentiation, Ig production, and other processes [46].

In this study, we found that the human epithelial cancer cells expressed two forms of IgM (membrane bound and secreted) as well as CD79A and CD79B. More important, the CD79 molecules were co-localized with the IgM to form a BCR-like complex. Moreover, as with BCR on the B cell surface [46], before stimulation with anti-human IgM, the BCR-like complex was uniformly distributed on epithelial cancer cells. In contrast, anti-IgM antibody cross-linked the BCR-like complex into small and large patches and induced a rapid Ca^++^ flux, and it further induced the activation of the PI3K and PLC-γ2 pathways, which may ultimately result in epithelial cancer cell proliferation.

It is well known that B cells can be stimulated by TLR9 ligand, the unmethylated CpG motif, to secrete IgM [21], [22], [47], [48], [49]. We found that CpG 2006, the most common TLR9 agonist, significantly increased secretion of IgM along with upregulated phosphorylation of ERK and IkB in the human epithelial cancer cells, similar to B cells. We were surprised that the two non-CpG ODN controls, CpG 2078 and GpC, which are considered to be unstimulatory controls for CpG 2006 in B cells, also induced IgM secretion in epithelial cancer cells in our experiments. Other researchers have discovered similar results. Merrell et al. found that not only CpG 2006 but also non-CpG ODN could promote the migration of epithelial cancer cells [32], [33]. Human and mouse non-B cells, have not evolved to recognize different CpG motifs in natural DNA. When a sequence-specific phosphorothioate-modified CpG dinucleotide is inverted to GC, some residual activity of the ODN is retained in a species-specific, TLR9-dependent manner [50], [51], [52], [53]. This may at least in part explain why CpG 2078 and GpC have stimulatory effects for IgM secretion in the epithelial cancer cells. Nevertheless, the facts that TLR9 agonists increases IgM secretion from cancer cells and that this increase can be abolished by chloroquine and MyD88 knockdown with siRNA suggest that the expression and secretion of cancer-derived IgM is mediated through the TLR9-MyD88 pathway.

B-1 B cells are characterized by the expression of the surface antigens CD20, CD27, as well as CD43, and play a key role in early protection against, and clearance of bacterial and viral infection via constitutive production of serum IgM referred to as “natural antibody”. Spontaneous, constitutive secretion of natural IgM accounts in large measure for the circulating protective natural antibody in both mice and humans. Most natural IgM are polyreactive, and can bind with low affinity to a number of different antigens, such as microbial pathogens and some autoantigens, contributing to early immunity before onset of the adaptive humoral response. Epithelial cells also play a essential role in early antimicrobial immunity. Recent evidence suggests that epithelial cells function not only as a physical barrier, but also as a regulator of innate and adaptive immune responses against foreign substances and microorganisms via helping to shape the responses of dendritic cells, T cells, and B cells, as well as to recruit inflammatory cells [54]. In particular, epithelial cells have been directly implicated in Th2 responses by serving as a critical interface between innate immune responses and Th2 immunity through the production of a group of epithelial cell-derived Th2-driving cytokines, including IL-25, IL-33, and thymic stromal lymphopoietin [55]. However, prior to our study, no evidence had suggested that epithelial cells are involved in the host defense via sharing with B-1 B cells the responsibility for natural IgM production.

In this study, we showed that many human epithelial cancer cells can spontaneously produce IgM without pathogenic infection. More important, like B-1 B cell-derived IgM, endothelial cell-derived IgM has natural IgM activity that recognizes well-defined antigens frequently used to analyze poly- and auto-reactivity of an antibody, such as ssDNA, dsDNA, LPS, and HEp-2 cell antigen. Our preliminary results showed that many mouse normal epithelial cells, such as liver, small intestine, and lung epithelial cells, can also produce IgM with natural antibody activity and that the secretion of this epithelial cell-derived IgM is increased upon encountering a bacterial infection (data not shown). These observations suggest that epithelial cells are involved in host defense via an unknown mechanism, sharing the responsibility for natural IgM production with B-1 B cells.

In addition, our observation that IgM was expressed at a higher level and frequency in human epithelial cancer cells than normal epithelia suggests that cancer-derived IgM may be involved in tumorigenesis. At least two possibilities for this observation exist. The first possibility is that IgM expressed by epithelial cancer cells directly controls the survival and proliferation of cancer cells, as supported by our finding that IgM heavy chain siRNA substantially induced apoptosis of cancer cell lines (data not shown). The second possibility is that cancer-derived IgM promotes the survival and proliferation of cancer cells via an innate immune-like mechanism. Environmental factors, such as infection and the resulting inflammation, are important regulators of tumor progression. The innate immune system can stimulate tumor development and progression through inflammation-dependent mechanisms. For example, chemokines and cytokines derived from immune and inflammatory cells can dramatically affect the host microenvironment and cancer cell behavior, resulting in increased growth and metastasis [56], [57]. As one of the most common causes of infection, some bacteria have been identified as causes of cancer [58]. In addition, bacterial DNA is the main infectious component of the bacteria. Synthetic ODN, especially those having unmethylated CpG motifs, can mimic the effects of bacterial DNA [23], [24], [25], [26]. Thus, our results suggest that infection might induce IgM secretion from non-B-cells and that this secreted IgM could promote an inflammation-like response via its activating complement and promoting opsonization effects, thereby becoming involved in tumor microenvironment formation.

Nevertheless, we should acknowledge that in this study, we just demonstrated that human epithelial cancer cells can produce IgM. Although the epithelial cancer cell-derived IgM may be involved in the innate immunity and tumor cell biology, till now, according to our results, we are not clear how this might impact these fields. Further studies need to be performed to reveal their functions and clinical significance.

In summary, we found for the first time that IgM is expressed in several types of non-B-cells, especially in epithelial cancer cells. TLR9 agonists stimulate the secretion of IgM by human epithelial cancer cells. Moreover, epithelial cancer cell-derived IgM has natural antibody activity.

## Supporting Information

Figure S1 **Different distribution between IgM and CD19 in human epithelial cancers.** Anti-human IgM (red) and anti-human CD19 (green) did not showed co-localization in human breast cancer tissues.
(TIF)

Figure S2 **Flow cytometry analysis showed lack of CD19 expression in human epithelial cancer cells.** Representative results of HeLa MR, HeLa, and HepG2 cells are shown. Raji was used as a positive control. Gray, isotype control; Black, monoclonal anti-human CD19-PE.
(TIF)

Table S1 **PCR primers used in this study.** PCR was performed to analyze the expression of Ig µ, Ig κ, CD79A, CD79B, TLR9 and MyD88 in the human epithelical cancer cell lines. And the primer sequences and PCR conditions were shown.
(DOC)

Table S2 **Realtime PCR primers used in the study.** Two-step realtime PCR was performed to quantify the expression of IgM and CD19 in the human epithelial cancer tissues using SYBR Green Master Mix. And the primer sequences were shown.
(DOC)

Table S3 **IgM expression in nonlymphocytic original tissues.** IgM expression was analyzed in tissue microarray of 202 tissue samples by immunohistochemistry, including cancer or normal tissues of epithelial, mesenchymal, and neuroglial origin as well as germ cells. And the staining results were shown.
(DOC)
