# Supplementary figures and images for: Spontaneous Production of Immunoglobulin M in Human Epithelial Cancer Cells
Source: PLoS One. 2012 Dec 12;7(12):e51423. doi: 10.1371/journal.pone.0051423 (PMC3520907; doi:10.1371/journal.pone.0051423)

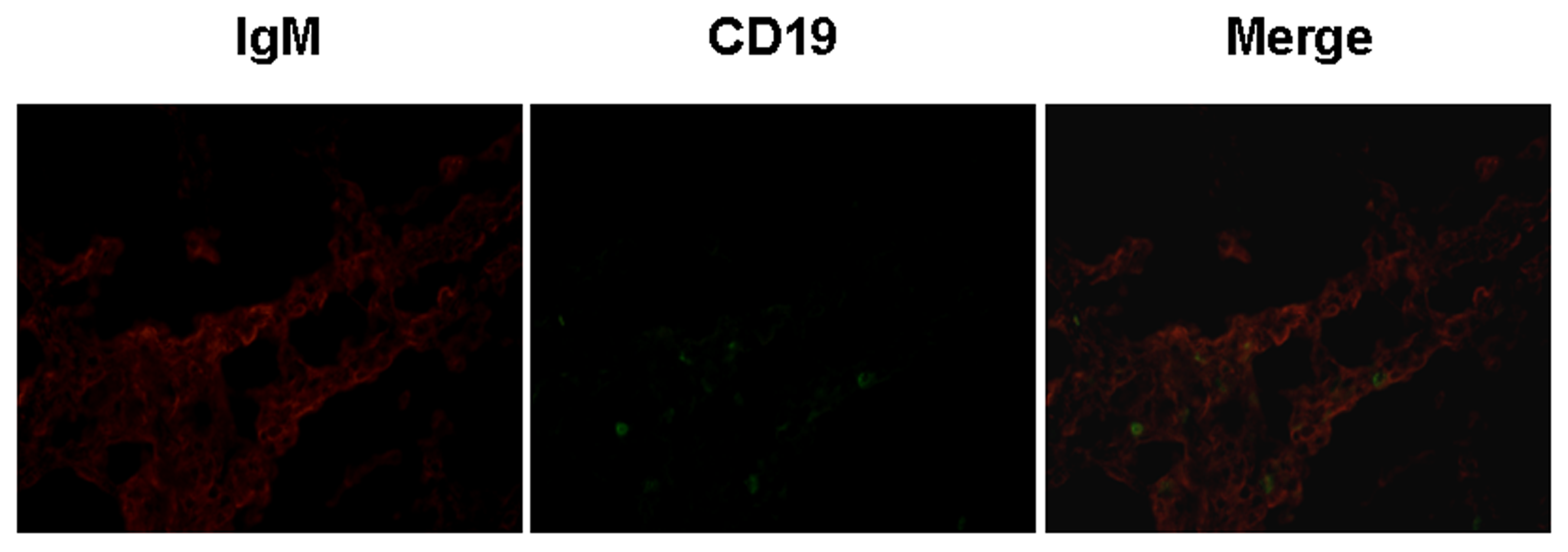

Supplement: Figure S1 — Different distribution between IgM and CD19 in human epithelial cancers. Anti-human IgM (red) and anti-human CD19 (green) did not showed co-localization in human breast cancer tissues. (TIF) [file pone.0051423.s001.tif]

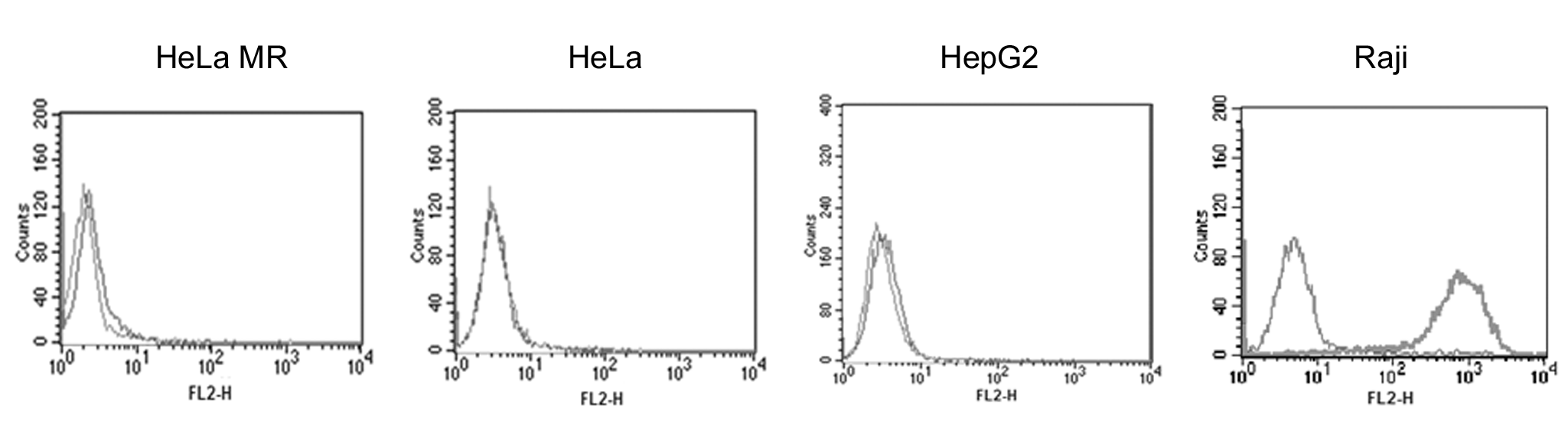

Supplement: Figure S2 — Flow cytometry analysis showed lack of CD19 expression in human epithelial cancer cells. Representative results of HeLa MR, HeLa, and HepG2 cells are shown. Raji was used as a positive control. Gray, isotype control; Black, monoclonal anti-human CD19-PE. (TIF) [file pone.0051423.s002.tif]
