# Supplementary material for: Spontaneous Production of Immunoglobulin M in Human Epithelial Cancer Cells
Source: PLoS One. 2012 Dec 12;7(12):e51423. doi: 10.1371/journal.pone.0051423 (PMC3520907; doi:10.1371/journal.pone.0051423)
Supplement: Table S1 — PCR primers used in this study. PCR was performed to analyze the expression of Ig µ, Ig κ, CD79A, CD79B, TLR9 and MyD88 in the human epithelical cancer cell lines. And the primer sequences and PCR conditions were shown. (DOC) [file pone.0051423.s003.doc]

**Table S1.** Primer sequences and PCR conditions.

**Target Primer sequences PCR conditions Cycles Product size**

Ig μ chain first-round 94°C, 30 seconds 40 457 bp

sense 5’-CCCGACTCCATCACTTTCTC-3’ 55°C, 45 seconds

antisense 5’-TGGTCTGCTTCAGTGGCG-3’ 72°C, 30 seconds

second-round 94°C, 30 seconds 25

sense 5’-GCTGAGGCAAAGGAGTCTG-3’ 55°C, 30 seconds

antisense same as first-round 72°C, 30 seconds

Ig κ chain first-round 94°C, 15 seconds 40 126-292 bp Sense Vκ1f/6 5’-TCAAGGTTCAGCGGCAGTGGATCTG-3’ 56°C, 30 seconds

Vκ2f 5’-GGCCTCCATCTCCTGCA GGTCTAGTC-3’ 72°C, 30 seconds

Vκ3f 5’-CCCAGGCTCCTCATCTATGATGCATCC-3’

Vκ4 5’-CA ACTGCAAGTCCAGCCAGAGTGTTTT-3’

Vκ5 5’-CCTGCAAAGCCAGCCAAGA CATTGAT-3’

Vκ7 5’-GACCGATTTCACCCTCACAATTAATCC-3’

antisense 5’-GGCGTTATCCACCTTCCACTGTAC-3’

second-round 94°C, 15 seconds 25

sense same as first-round 56°C, 30 seconds

antisense Jκ1-4 5’-CTTACGTTTGATCTCCACCTTGGTCCC-3’ 72°C, 30 seconds

Jκ5 5’-CTTACGTTTAATCTCCAGTCGTGTCCC-3’

CD79A sense 5’-CTGCTGCAACTCAA A CTAACCAA-3’ 94°C, 30 seconds 40 789 bp

antisense 5’-CCAGGGAAGTGAGCTGAGACA-3’ 58°C, 30 seconds 675 bp

72°C, 40 seconds

CD79B sense 5’-CGGACGTTGTCACGGGTTTG-3’ 94°C, 30 seconds 40 933 bp

antisense 5’-TGCCCT GTTGTCCTTCTACTCC-3’ 58°C, 30 seconds 621 bp

72°C, 40 seconds

TLR9 sense 5’-GCCAGACCCTCTGGAGAA-3’ 94°C , 1 minute 40 450 bp

antisense 5’-GGCACAGTCATGATGTTGTTGTA-3’ 56°C, 1 minute

72°C, 1 minute

MyD88 sense 5’-GCCGCCTGTCTCTGTTCTTG-3’ 94°C, 1 minute 40 472 bp

antisense 5’-CGATAGTTTGTCTGTTCCAGTTGC-3’ 56°C, 1 minute

72°C, 1 minute
