# Supplementary material for: Spontaneous Production of Immunoglobulin M in Human Epithelial Cancer Cells
Source: PLoS One. 2012 Dec 12;7(12):e51423. doi: 10.1371/journal.pone.0051423 (PMC3520907; doi:10.1371/journal.pone.0051423)
Supplement: Table S2 — Realtime PCR primers used in the study. Two-step realtime PCR was performed to quantify the expression of IgM and CD19 in the human epithelial cancer tissues using SYBR Green Master Mix. And the primer sequences were shown. (DOC) [file pone.0051423.s004.doc]

**Table S2.** Realtime PCR primers used in the study.

| **Genes** | **Sense primer (5**′**-3**′**)** | **Antisense primer (5**′**-3**′**)** |
| --- | --- | --- |
| GAPDH  IgM  CD19 | AAGGTGAAGGTCGG AGTCAA  GCTGAGGCAAAGGAGTCTG  TCAGCTGTGACTTTGGCTTATCTG | AATGAAGGGGTCATTGATGG  TGGTCTGCTTCAGTGGCG  AGTCATTCGCTTTCTTTTCCT |
