# Supplementary material for: Spontaneous Production of Immunoglobulin M in Human Epithelial Cancer Cells
Source: PLoS One. 2012 Dec 12;7(12):e51423. doi: 10.1371/journal.pone.0051423 (PMC3520907; doi:10.1371/journal.pone.0051423)
Supplement: Table S3 — IgM expression in nonlymphocytic original tissues. IgM expression was analyzed in tissue microarray of 202 tissue samples by immunohistochemistry, including cancer or normal tissues of epithelial, mesenchymal, and neuroglial origin as well as germ cells. And the staining results were shown. (DOC) [file pone.0051423.s005.doc]

**Table S3. IgM expression in nonlymphocytic original tissues.**

| **Tissue origin** | **Grade** | **Tissue type** |  |  |  | **No.** | **No.** | **%** |
| --- | --- | --- | --- | --- | --- | --- | --- | --- |
|  |  | | **-** | **+** | **++** | **Cases** | **Positive** | **Positive** |
| **Epithelium** | **Malignant** | |  |  |  | **34** | **17** | **50.0%** |
|  |  | Breast | 2 | 2 | 1 | 5 | 3 |  |
|  |  | Esophagus | 1 | 1 |  | 2 | 1 |  |
|  |  | Stomach | 2 | 1 |  | 3 | 1 |  |
|  |  | Colon | 1 | 1 |  | 2 | 1 |  |
|  |  | Liver |  | 1 | 1 | 2 | 2 |  |
|  |  | Lung | 2 | 3 |  | 5 | 3 |  |
|  |  | Kidney | 3 |  |  | 3 | 0 |  |
|  |  | Pancreas | 1 | 2 |  | 3 | 2 |  |
|  |  | Rhinopharynx | 2 |  |  | 2 | 0 |  |
|  |  | Prostate |  | 1 |  | 1 | 1 |  |
|  |  | Cervix | 1 | 1 |  | 2 | 1 |  |
|  |  | Uterus | 1 | 1 |  | 2 | 1 |  |
|  |  | Ovary | 1 |  | 1 | 2 | 1 |  |
|  | **Nonmalignant** | |  |  |  | **66** | **23** | **34.8%** |
|  |  | Breast | 5 | 4 |  | 9 | 4 |  |
|  |  | Esophagus | 3 | 2 |  | 5 | 2 |  |
|  |  | Stomach | 2 | 2 |  | 4 | 2 |  |
|  |  | Colon | 7 | 2 |  | 9 | 2 |  |
|  |  | Liver | 5 | 2 | 3 | 10 | 5 |  |
|  |  | Lung | 7 | 2 |  | 9 | 2 |  |
|  |  | Kidney | 6 | 1 |  | 7 | 1 |  |
|  |  | Pancreas | 5 | 1 |  | 6 | 1 |  |
|  |  | Prostate |  | 1 |  | 1 | 1 |  |
|  |  | Cervix | 2 | 1 |  | 3 | 1 |  |
|  |  | Uterus |  | 2 |  | 2 | 2 |  |
|  |  | Ovary | 1 |  |  | 1 | 0 |  |
| **Mesenchyma** | **Malignant** | |  |  |  | **47** | **1** | **2.1%** |
|  |  | Striated muscle | 9 |  |  | 9 | 0 |  |
|  |  | Smooth muscle | 11 |  |  | 11 | 0 |  |
|  |  | Fibrous tissue | 3 |  |  | 3 | 0 |  |
|  |  | Fatty tissue | 17 | 1 |  | 18 | 1 |  |
|  |  | membrane  Blood vessel | 3  3 |  |  | 3  3 | 0  0 |  |
|  | **Nonmalignant** | |  |  |  | **34** | **1** | **2.9%** |
|  |  | Striated muscle | 9 | 1 |  | 10 | 1 |  |
|  |  | Smooth muscle | 8 |  |  | 8 | 0 |  |
|  |  | Heart muscle | 5 |  |  | 5 | 0 |  |
|  |  | Fibrous tissue | 4 |  |  | 4 | 0 |  |
|  |  | Fatty tissue | 4 |  |  | 4 | 0 |  |
|  |  | Blood vessel | 3 |  |  | 3 | 0 |  |
| **Nerve** | **Malignant** |  |  |  |  | **13** | **0** | **0%** |
|  |  | Gliocyte | 12 |  |  | 12 | 0 |  |
|  |  | Meninges | 1 |  |  | 1 | 0 |  |
| **Testis** | **Malignant** | Seminoma |  | **2** |  | **2** | **2** | **100.0%** |
|  | **Nonmalignant** | **Spermatocyte** |  | **2** |  | **2** | **2** | **100.0%** |
| **B lymphoid** |  |  | **1** | **1** |  | **2** | **1** | **50.0%** |
| **T lymphocyte** |  |  | **2** |  |  | **2** | **0** | **0%** |

1. -, >95% cells were negative; +, 5%–25% cells were positive; ++, >25% cells were positive.
2. The positive rate of malignant epithelial tissues *vs.* that of nonmalignant epithelial tissues: *P* = 0.1429.
3. The positive rate of epithelial tissues *vs.* that of mesenchymal tissues: *P* < 0.0001.
